# Post-transplant infections: An ounce of prevention

**DOI:** 10.4103/0971-4065.73431

**Published:** 2010-10

**Authors:** V. Jha

**Affiliations:** Department of Nephrology, Postgraduate Institute of Medical Education and Research, Chandigarh, India

**Keywords:** Infections, kidney transplantation, prevention

## Abstract

Infections are the leading cause of hospitalization in transplant recipients. The increased risk of new onset diabetes after transplantation, cardiovascular disease, post-transplant lymphoproliferative disorders adversely affects allograft outcomes. Risk is determined by epidemiologic exposure, immunosuppressive therapy and prophylaxis. The predictable sequence of appearance of infections helps in making management decisions. High likelihood of infections with unusual and multiple organisms necessitates aggressive use of imaging techniques and invasive procedures. Serologic tests depend upon antibody response and are unreliable. Nucleic acid based assays are sensitive, rapid, and allow detection of subclinical infection and assessment of response to therapy. Preventive steps include screening of donors and recipients and vaccination. All indicated vaccines should be administered before transplantation. Inactivated vaccines can be administered after transplantation but produce weak and transient antibody response. Boosters may be required once antibody titers wane. Post-transplant chemoprophylaxis includes cotrimoxazole for preventing urinary tract infections, pneumocystis and *Nocardia* infections; ganciclovir, valganciclovir, or acyclovir for cytomegalovirus related complications in at-risk recipients; and lamivudine for prevention of progressive liver disease in HBsAg positive recipients. Viral load monitoring and pre-emptive treatment is used for BK virus infection. Infection with new organisms has recently been reported, mostly due to inadvertent transmission via the donor organ.

## Introduction

Optimal use of immunosuppressive drugs in a renal transplant recipient (RTR) requires a careful balancing act. Availability of potent and specific immunosuppressive agents has reduced the incidence of acute rejection to about 10–15% in most centers. However, despite refinements in diagnostic techniques and discovery of new anti-microbial drugs, the risk of infection amongst transplant recipients has not come down.[1] About 70% of all RTRs experience at least one infection episode by 3 years. The 2008 USRDS report[2] showed increase in hospitalization rates for infection from 5.9% per 100 patient years in 2001–2003 to 6.5% per 100 patient years in 2004–2006; hospitalization rates for other causes decreased over the same period. Infections are responsible for 18% of all deaths with functioning grafts in the US, and are the leading cause of death in the developing countries.

Infection risk is even greater in the pediatric transplant population. Data from the North American Pediatric Renal Transplant Cooperative study show that 38–42% patients transplanted between 1987 and 2002 required hospitalization for infections. Infection was the primary cause of hospitalization in the first 2 years after transplantation, exceeding that for rejection.[3] The frequency of admissions due to infections during the first 6 months after transplantation remained unchanged over time, but increased in the 6–24 month period in patients of more recent vintage. Infections also increase the risk of new onset diabetes after transplantation (NODAT), cardiovascular events, post-transplant lymphoproliferative disorders (PTLD) and adversely affect allograft outcomes.

Bacterial infections are approximately twice as frequent as viral infections in RTR. About 13% of all patients transplanted between 1996 and 2000 in the US required hospitalization for bacterial infections in the first 3 years compared to 6% for viral infections.[4] Vascular access and urinary tract infections (UTIs) were the most frequent bacterial infections, whereas cytomegalovirus (CMV) was the commonest viral infection. Extremes of recipient age, female gender, deceased donor source, older donor age, CMV+ve donor, time on dialysis and systemic lupus erythematosus (SLE) as the cause of kidney disease increased the infection risk.[4]

The infection risk at any given time after transplant is determined by the overall balance between the nature and intensity of epidemiologic exposure, net status of immunosuppression and the current nature of protection as determined by the vaccination and chemoprophylaxis status. Evaluation of exposure requires obtaining a history of travel to areas where certain infections may be endemic, dietary habits (e.g., cryptosporidium from well water and *Salmonella* and *Lisetria* from uncooked meat or dairy products), and details regarding work and hobbies (*Aspergillus* from construction sites, saprophytic fungi from gardening and leptospirosis in field workers). The overall status of immunosuppression is determined by complex and dynamic interactions between the recipient (age, gender, genetic background, underlying clinical condition), the transplanted organ and drugs. It is also affected by other complications such as a breach in the integrity of muco-cutaneous barriers, leukopenia, NODAT, poor graft function, liver dysfunction and malnutrition.[5]

No consistent relationship has been shown between a specific immunosuppressive agent and overall infection risk. Mycophenolate mofetil (MMF) has been linked to an overall increase in infections, especially viral,[4] and antilymphocyte antibody to CMV reactivation.[6] Higher incidence of BKV nephropathy has been noted amongst those on the “potent” combination of tacrolimus and MMF.

The right level of immunosuppression that affords protection against rejection while minimizing infection risk is achieved in clinical practice by trial and error, based on monitoring of drug levels, leukocyte counts and surveillance for metabolic complications. Studies on evaluation of biomarkers for immune monitoring have focused toward identification of rejection.[7] No reliable method exists currently for objective evaluation of net status of immune system to predict infection risk.

Attempts to develop such a measure have relied on determination of the functional status of T lymphocytes. The Cylex ImmuKnow assay measures the ability of T lymphocytes to respond to non-specific immunostimulation with phytohemagglutinin by producing ATP. Response is quantified in terms of the amount of ATP released in the supernatant. In one study,[8] recipients with ImmuKnow values of 25 ng/ml were 12 times more likely to develop an infection compared to those with a stronger response. Values ≥700 ng/ml conferred a 30-fold increase in rejection risk. RTR with BK viremia showed lower ImmuKnow values in comparison to BKV negative recipients.[9] Serial studies in patients with viral infection have shown increase in values along with viral clearance following reduction of immunosuppression.[10] This test has been cleared by US Food and Drug Administration (FDA) for immune cell function monitoring in immunosuppressed patients. Its value, however, needs to be determined in prospective studies. Recently, an association was shown in a cohort of heart transplant recipients between low circulating levels of soluble CD30, a cell-surface marker expressed by a subset of memory T cells, and infection.[11]

## General Considerations in Diagnosis and Management of Infections in RTR

**Figure 1:**
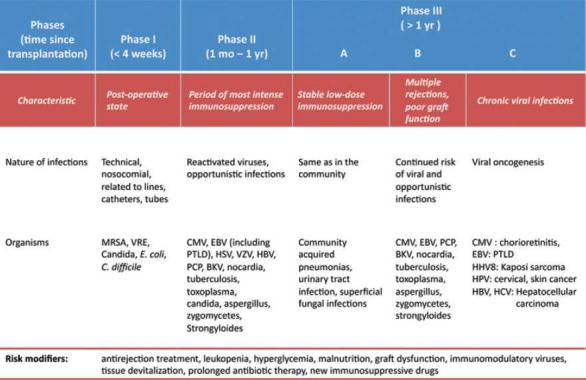
The phases in the “timetable of infections” according to time elapsed since transplantation and the risk status of the patient. The risk status changes in any stage if any of the modifiers are present

The broadly predictable pattern of the nature of infections encountered following transplantation gave rise to the concept of a “timetable of infections” that divides the risk period into three overlapping zones [Figure 1]. The table helps in making informed decisions about the likely nature of infections and tailoring of diagnostic and therapeutic resources.[5]

The possibility of infection needs to be considered in all febrile presentations of RTR. Fever may occasionally be absent, and symptoms may solely be related to one or more organ systems. The presentation may be different in RTR compared to the general population. For example, parvovirus B19 infection presents as pure red cell aplasia in this group, in contrast to erythema infectiosum in immunocompetent individuals. BK polyoma virus infection, asymptomatic in general population, causes renal allograft dysfunction.

The possibility of infections with unusual, often exotic, organisms and the high likelihood of polymicrobial infections necessitate a multidisciplinary approach with involvement of other specialists including the ID team. Early and aggressive use of imaging techniques such as ultrasound, computed tomography (CT) scans or magnetic resonance imaging (MRI), and invasive procedures like bronchoalveolar lavage, imaging guided aspiration and/or biopsies for obtaining specimens for histological and/or microbiological examination are essential for accurate diagnosis.

Serologic tests are of limited value since antibody response is attenuated in the immunocompromised host. Quantitative nucleic acid based assays are sensitive, quick, and useful for detection of subclinical infection, assessing response to therapy and identifying drug resistance. Studies have documented the adverse impact of subclinical CMV and Epstein–Barr virus (EBV) viremia on graft function.[12] Viral load monitoring is used to guide therapy; failure to clear the virus is associated with strong risk of recurrence of CMV disease.[13] Multiplex assays allow simultaneous quantitative determination of several microorganisms including CMV, EBV, human herpesvirus (HHV)-6 and BK virus and a number of fungi. The main problem with these assays is lack of reproducibility across different laboratories.

The non-specific nature of presentation often necessitates the initiation of broad-spectrum therapy before a specific etiologic diagnosis can be made. Development of CMV or EBV disease indicates over immunosuppression and should prompt reduction in immunosuppressive drug dosage.

## Prevention of Post-transplant Infections

Adoption of preventive strategies has considerably reduced the burden of infection in RTR. This process starts before transplantation with pre-transplant screening of donors and recipients, avoidance of use of blood products, use of leukocyte filters during transfusions, treatment of pre-existing infections, immunoprophylaxis (vaccination), and continues after transplantation with tailored chemoprophylaxis and surveillance.

Donor screening is aimed at preventing transmission of latent infections including locally prevalent ones, e.g., tuberculosis and schistosomiasis, via the infected organ.[14] Organs from with hepatitis (B or C) (HBV or HBC) or HIV infected donors are not used for transplant. Recently, some centers have started using organs from HCV or HIV positive donors for recipients who already harbor these infections after informed consent is obtained.[15] A recent analysis showed that the adjusted hazard ratio for death among HCV positive recipients of kidneys from HCV antibody positive donors was lower compared to those who remained on dialysis.[16] HBV core antibody-positivity indicates a low risk of transmission, and kidneys from these donors can be used in HBV antibody-positive recipients.[17,18]

Issues related to timing (if performed during the window period, i.e., between the infection and seroconversion) host (poor antibody response in the immunocompromised patient with end stage renal disease) or organism (genetic change, e.g., HBV precore mutant) can result in a false negative serologic test. Nucleic acid based assays are not subject to these errors. Uncommon pathogens for which screening is not performed routinely (rabies, SARS, West Nile virus) can be transmitted through a contaminated allograft.

## Vaccination

Recommendations for vaccination in transplant recipients are based largely on data from general population. Vaccination status should be reviewed at initial evaluation of chronic kidney disease (CKD), and all vaccinations recommended for the general population should be administered. Pediatric CKD patients should be vaccinated against varicella, influenza, hepatitis B and *Pneumococcus*. Vaccines should be administered early to CKD patients, since poor immune memory in advanced stages of CKD and after transplantation results in weak antibody response.[19] Pre-transplant vaccination may not be feasible in children and in areas with limited dialysis facilities, necessitating post-transplant vaccination. Experts agree that inactivated vaccines are safe when administered after transplantation. Use of live vaccines, however, is controversial. A couple of studies[20,21] demonstrated the safety of varicella and measles vaccines in small number of patients after transplantation, but the balance of opinion suggests that the risks of live vaccines outweigh potential benefits and hence should not be used.[22]

Data on the clinical efficacy of individual vaccines is limited. Observational studies have documented the salutary effect of pre-transplant vaccination on the course of varicella infection after transplantation.[23,24] Post-transplant influenza and pneumococcal vaccinations lead to protective antibody titers in a majority of RTRs.[19] The antibody response is weak for post-transplant hepatitis B vaccine. Antibody titers should be monitored with booster vaccination once the titers fall below 10 IU/ml.

The American Society of Transplantation[22] suggests delaying resumption of vaccinations after transplantation until the immunosuppressive drug dosage has been reduced to the lowest maintenance levels and documentation of vaccine efficacy by serologic assays. There is no consensus on the frequency of monitoring; annual verification is sufficient in most instances. Vaccination is desirable for pathogens that may be encountered while traveling to endemic areas as long as the recommended vaccinations are inactivated. Recommended vaccines in transplant candidates and recipients are shown in Table 1.

**Table 1:** Recommended vaccines for renal transplant recipients

Vaccine	Monitoring required?
Can be given before/after transplantation	
Influenza	No
Hepatitis B	Yes
Hepatitis A	Yes
Inactivated polio	No
Pneumococcal polysaccharide vaccine	Yes
Meningococcus	No
Tetanus	No
Conjugated pneumococcal vaccine*	Yes
Pertussis^a^	No
Diphtheria^a^	No
*Haemophilus influenza*^a^	Yes
Japanese encephalitis^b^	Yes
*Salmonella typhi* Vi^b^	Yes
Rabies^c^	No
Should be given only before transplantation	
BCG	No
Varicella	No
Measles^a^	Yes
Mumps^a^	Yes
Rubella^a^	Yes

* For children <2 years of age

a Recommended only for pediatric recipients

b Recommended if traveling to an endemic area

c Recommended in case of exposure

## Chemoprophylaxis

Drugs provide effective protection against a variety of potential pathogens in RTR. One single strength tablet of cotrimoxazole protects against bacterial UTI, *Pneumocystis carinii* pneumonia (PCP), *Toxoplasma, Listeria* and Nocardia. It is be used for 6–12 months after transplantation, the period of maximum risk for PCP and Nocardia, and graft pyelonephritis, bacteremia and poor graft function following UTI.[25] The risk of UTIs increases after stoppage of prophylaxis, but late infections are usually benign.[26] Ciprofloxacin also provides effective prophylaxis for UTI and cotrimoxazole protects against PCP even when taken three times a week, but once a day cotrimoxazole is preferred due to its convenience.

## Cytomegalovirus

CMV impacts the course of RTR in several ways. CMV disease presents with a “flu-like” illness, with or without tissue invasion, manifested as bone marrow suppression, hepatitis, colitis, interstitial pneumonia or CNS involvement. Through its immunomodulatory properties, CMV infection also increases the risk of invasion by opportunistic organisms and allograft rejection.[5,6]

Without prophylaxis, 10–60% of RTR develop CMV disease, but risk is not equal in all. Risk stratification is on the basis of recipient and donor CMV serostatus at the time of transplantation.[6] Seronegative recipients who receive organs from seropositive donors (D+R–) have a 40–50% chance of developing the disease. Endogenous reactivation leading to CMV disease occurs in 10–15% of seropositive recipients (D+/–R+). The figure may be higher in those who receive antilymphocyte therapy. The risk is negligible in with D–R– transplants.

Systematic reviews have shown that prophylaxis with oral or intravenous ganciclovir, valganciclovir, acyclovir or valacyclovir reduces the incidence of CMV disease, CMV-associated mortality, all cause mortality and clinically important opportunistic infections.[27,28] One analysis found that prophylaxis significantly reduced the rate of graft rejection,[28] but the other did not.[27] Oral valganciclovir and intravenous ganciclovir are equally efficacious in preventing CMV infection and disease.[29] Similarly, oral and intravenous ganciclovir yielded similar results. Ease of administration makes oral valganciclovir the preferred agent. The recommended dose is 900 mg/d, but recent studies have shown that 450 mg/d is also effective for prevention.[30,31] Prophylaxis also reduces the risk of herpes simplex and zoster disease.[27] Acyclovir is less effective and should be restricted to situations where ganciclovir/valganciclovir cannot be used due to economic reasons.

The exact duration of prophylaxis is not clear. The current recommendations suggest 3 months,[6,32] extended to 6 months in those receiving antilymphocyte induction. A recent meta-analysis did not find a difference in outcomes whether the treatment was for less or more than 6 weeks. A recently recognized effect of prophylaxis has been to delay the onset of CMV disease. Over 90% of disease in patients who receive prophylaxis is now seen after 90 days. Late onset disease is an independent predictor of mortality and graft loss.[33,34] Widespread prophylaxis also carries the risk of development of resistance. In a recent study,[35] 15% of late onset disease was due to drug resistant strains.

An alternative approach of CMV disease prevention is pre-emptive therapy that relies upon CMV viral load monitoring and institution of treatment with ganciclovir at a predetermined threshold.[36] The potential advantages of this strategy are its cost-effectiveness and avoidance of potential toxicities of antiviral agents. An randomized clinical trial[37] showed this approach to be as effective as routine prophylaxis in preventing disease, but without any cost advantage, probably because of the added cost of monitoring. A recent study, however, found pre-emptive therapy to be inferior to universal prophylaxis in terms of rejection risk and preservation of renal function.[38] On balance, therefore, the pre-emptive approach has been suggested to be restricted to low risk (D–/R) recipients. Use of antilymphocyte antibody therapy for acute rejection increases the risk of CMV disease, which can be effectively prevented by use of ganciclovir.[39,40]

## Epstein–Barr Virus

The most important clinical consequence of EBV infection in RTR is development of PTLD. The risk is highest in recipients who are EBV-antibody negative at the time of transplantation. A case-control study showed that ganciclovir and acyclovir were effective in preventing PTLD,[41] possibly through suppressing subclinical EBV viremia.[12] Data from the Collaborative Transplant Registry, however, did not confirm this finding, but showed a protective effect of anti-CMV immunoglobulin.[42]

## Hepatitis B

HBsAg positive RTRs have a high likelihood of developing chronic liver disease.[43] Lamivudine, a cytosine analog started at the time of transplantation, stabilizes liver function. A meta-analysis[44] showed alanine transaminase (ALT) normalization, and HBV-DNA and HBeAg clearance with lamivudine prophylaxis in 81, 91 and 27%, respectively, of HBsAg positive RTRs. Recipients not on lamivudine experienced deterioration in liver enzymes and increasing HBV DNA levels within a few months, necessitating initiation of therapy.[45] The benefits are directly proportional to the duration of prophylaxis. Withdrawal can lead to increased viral replication and relapse of liver disease, even resulting in liver failure. Development of resistance, reflected by a secondary increase in the HBV DNA titers, is the major risk of long-term use. The optimal duration that ensures long-term remission of viremia, maintenance of normal liver function and minimizes the development of resistance remains unclear. Newer antiviral agents like adefovir and entecavir are effective in those with lamivudine resistance.[46,47] Whether substitution of lamivudine with entecavir for primary prophylaxis will prevent development of resistance is currently unknown.

## BK Virus

BK virus, a member of polyoma group of viruses, is ubiquitous in humans. After primary infection, it establishes latency in the urothelium. Reactivation occurs in the setting of immunosuppression, leading to clinically insignificant urinary shedding in 30–60% of RTR.[48] The infection disseminates in about 5–10% and produces the syndrome of BK virus nephropathy (BKN). It is seen as cytopathic changes in renal tubules and interstitial infiltration along with a positive staining for SV40 antigen on allograft biopsy. Over 50% grafts are lost in the first year after diagnosis despite reduction in immunosuppression and treatment with cidofovir and/or leflunomide.[49] Histological changes may not be always obvious because of the focal nature of the disease.[50] Viral load monitoring can identify patients at risk of allograft damage. Demonstration of >10^4^ copies of virus in plasma or >10^7^ copies in urine identifies those likely to develop BKN.[48,51,52] Three-monthly screening is recommended; any high value should be reconfirmed within 3 weeks.[53] Serial monitoring is also useful in following patient after reduction of immunosuppression and in those being considered for re-transplantation to document resolution.

## Antifungal Prophylaxis

The risk of *Candida* infection as a result of increased oral colonization is heightened in the early post-transplant period, during periods of intensified immunosuppression such as after treatment for rejections or after prolonged courses of broad-spectrum antibiotics. Prophylactic topical antifungals such as nystatin or clotrimazole help eradicate the colonization without producing systemic adverse effects.

## Emerging Infections in Transplantation

Recent years have seen the identification of disease due to a number of organisms hitherto not seen in RTR [Table 2][54,55] Clinical syndrome may be a result of primary infection due to transmission via the donor organ or following environmental exposure, or secondary to reactivation of latent infection following immunosuppression. Infection may be asymptomatic, or present with either mild self-limiting febrile illness or severe multisystem disease. Diagnosis is usually made by molecular techniques. Treatment includes lowering of immunosuppression and use of intravenous immunoglobulin or antiviral agents.

**Table 2:** Emerging infections in transplant recipients

Pathogen	Mode of transmission	Usual time of presentation	Presenting features	Diagnosis	Treatment
HHV-6	Reactivation of latent infection	Commonest in first 2–4 weeks, may occur up to 2 years	Fever, rash, myelosuppression, hepatitis, pneumonitis, encephalitis ↑ risk of CMV and opportunistic infections	PCR	Ganciclovir
HHV-7	Transmission from donor			Histopathology	Cidofovir Foscarnate
Adenovirus	Reactivation, nosocomial transmission	Commonest in first 3 months, may occur until several years	Interstitial nephritis, hemorrhagic cystitis, pneumonitis	Immunohistochemistry PCR in plasma	IVIG Cidofovir
West Nile virus	Transmission from donor,blood transfusion, environmental exposure		Fever, meningoencephalitis,hyporeflexic paralysis	PCR (short viremic phase)Serology (may be delayed) IgM antibody in CSF	IVIG
LCM	Transmission from donor,	First 4 weeks	Fever, diarrhea, asepticmeningitis, interstitial pneumonia, hepatitis, multisystem failure	Cerebrospinal fluid PCR, serology	
Parainfluenza and metapneumovirus	Environmental and nosocomial transmission	After 1 year illness, pneumonia	Fever, upper respiratory	PCR Antigen detection on respiratory secretions	Ribavirin
Parvovirus B19	Transmission from donor	First year	Fever, joint pain, pure redcell aplasia, hepatitis, pneumonitis	PCR Bone marrow examination	IVIG
Respiratory syncytial virus	Nosocomial transmission	Any time	Upper respiratory tract infection, interstitial pneumonia	PCR Antigen testing on respiratory secretions	Ribacirin IVIG
Rotavirus	Environmental transmission	Any time	Self-limiting diarrhea, lowergastrointestinal bleeding		None

HHV: human herpesvirus; LCM: lymphocyte choriomeningitis virus, CSF: cerebrospinal fl uid, IVIG: intravenous immunoglobulin; PCR: polymerase chain reaction

In conclusion, infections remain a major problem in the transplant population. They are a main cause of death with functioning graft, and cause a number of other complications that increase morbidity. Molecular diagnostic techniques have allowed earlier identification and better monitoring of infections. Prophylactic strategies include vaccination and targeted post-transplant chemoprophylaxis. Use of drugs carries the risk of late and resistant infections. A high index of suspicion and early and aggressive use of diagnostic techniques are essential for accurate diagnosis and improved outcomes.
